# Collapse of Corroded Pipelines under Combined Tension and External Pressure

**DOI:** 10.1371/journal.pone.0154314

**Published:** 2016-04-25

**Authors:** Hao Ye, Sunting Yan, Zhijiang Jin

**Affiliations:** Institute of Process Equipment, College of Chemical and Biological Engineering, Zhejiang University, Hangzhou, China; University of Zaragoza, SPAIN

## Abstract

In this work, collapse of corroded pipeline under combined external pressure and tension is investigated through numerical method. Axially uniform corrosion with symmetric imperfections is firstly considered. After verifying with existing experimental results, the finite element model is used to study the effect of tension on collapse pressure. An extensive parametric study is carried out using Python script and FORTRAN subroutine to investigate the influence of geometric parameters on the collapse behavior under combined loads. The results are used to develop an empirical equation for estimating the collapse pressure under tension. In addition, the effects of loading path, initial imperfection length, yielding anisotropy and corrosion defect length on the collapse behavior are also investigated. It is found that tension has a significant influence on collapse pressure of corroded pipelines. Loading path and anisotropic yielding are also important factors affecting the collapse behavior. For pipelines with relatively long corrosion defect, axially uniform corrosion models could be used to estimate the collapse pressure.

## 1. Introduction

Pipelines and risers are among the most important equipment in subsea oil and gas exploration. As the offshore activities go into deeper water, they are inevitable to suffer high hydrostatic pressure as well as bending and tension during installation and operation. Considering the fact that it is uneconomical or even difficult to repair pipelines in deep sea, failure is not allowed to occur. Collapse could be the most important design factor under high external pressure.

Collapse of circular shells under single or combined loads has been widely investigated in the past few decades. Timoshenko and Gere [1] studied the elastic collapse of infinite long tube under external pressure and derived the Eigenvalue buckling solution of perfect shell. As to the tube with initial ovality, they suggested the collapse pressure to be the one to cause initial yielding. The collapse pressure determined in this way is always smaller than the real value and should serve as a conservative estimation, but it shows that the collapse pressure is strongly influenced by initial imperfection.

Timoshenko’s results are limited to infinite long shells, the boundary conditions of which were neglected. Pinna and Ronalds [2] examined the eigenvalue buckling solution of shorter shells under external pressure and gave an equation applicable to various boundary conditions.

For deep-water pipelines, the pipelines’ diameter-to-thickness ratio (D/t) is lower and material plasticity will influence the collapse behavior. Yeh and Kyriakides [3] studied the collapse of infinite long pipeline under external pressure with initial ovality and wall thickness variation. They showed that D/t, material property and initial ovality (Δ_0_) affected collapse pressure significantly and wall thickness variation had a smaller effect, while yield anisotropy could affect collapse pressure positively or negatively. Similar conclusions could be found in [4–6]. Based on finite element analyses, He et al. [7] gave a simple equation to predict the collapse pressure of thick-walled pipelines.

Offshore pipelines can experience combined external pressure, tension and bending [8, 9]. The behavior on collapse response and the collapse loads were found to be influenced by loading path and hardening rule as well as material properties [10]. Asymmetric collapse modes were identified in experiments in [11] and the causes of this phenomenon were the orientation of initial ovality, residual stress and wall thickness variation [11, 12]. Bai et al. [13] studied the response of thick pipelines under combined loads by using ABAQUS [14] and proposed some equations for predicting the collapse loads.

Although many efforts have been made to protect offshore pipeline, corrosion may still occur due to losing of protective coating which reduces the resistance to collapse. While the collapse strength of corroded pipeline is an important issue, only few works have been done on it. Fatt [15] and Xue [16–18] extended Timoshenko’s method and derived solutions of elastic collapse of infinite long, thin-walled pipeline under external pressure with corrosion defect running through the full length. Netto [19–21] took a further look at collapse of thicker pipelines under external pressure with corrosion defects running through part of the length and developed an empirical equation to estimate the collapse pressure. Sakakibara [22] investigated the collapse of axially uniform corroded pipelines under external pressure through experimental and numerical methods. Yet the studies on collapse behavior of corroded pipelines under combined loads are limited.

This paper aims at investigating the collapse behavior of corroded pipelines under combined external pressure and tension numerically. The numerical model is verified by comparing the numerical results with existing experimental results. Then the programming language Python and FORTRAN are used to carry out extensive parametric studies to study the influence of diameter-to-thickness ratio, initial ovality, defect geometries and ovality orientation on collapse pressure under combined loads. Results of parametric studies are used to develop an empirical equation for predicting the collapse pressure under tension. Later, the influence of corrosion length, loading path, and initial imperfection length and yield anisotropy on the collapse behavior has also been studied.

## 2. Finite Element Modeling and Model Verification

### 2.1 Geometry

**Fig 1:**
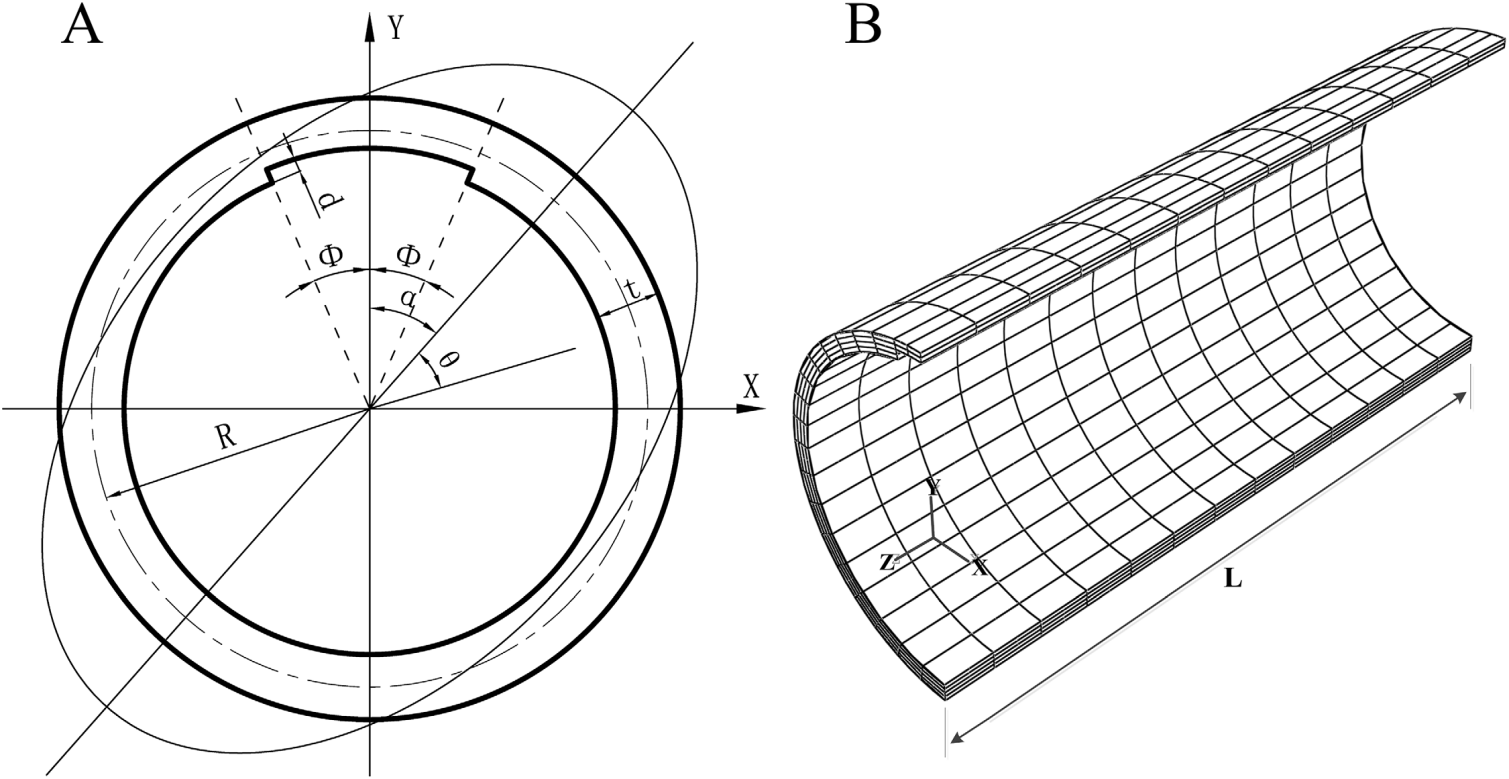
Geometry and mesh for axially uniform corroded pipeline. (A) cross-section of corroded pipeline. (B) Finite element model of the corroded pipeline.

For pipelines with a long longitudinal corrosion defect, a section of axially uniform corroded pipeline is modeled by ABAQUS and is discretized with quadratic element with reduced integration (C3D20R) in all analyses, see Fig 1. It should be mentioned that the actual corrosion defect is in arbitrary shape and the assumed groove shape defect will result in conservative estimation. Axially uniform initial ovality is taken as the form of initial imperfection. In order to verify with existing experimental results [22], symmetric collapse modes are studied in this section. The initial ovality is assumed to be symmetric about YZ plane with its major axis coincides with Y-axis (i.e. α = 0°) and could be defined by radial displacement (w) as w = Δ_0_Rcos2θ, where R is the radius of mid-plane and Δ_0_ is defined as:

$$\Delta_{0}=\frac{Dmax-Dmin}{Dmax+Dmin}$$ (1)

D_max_ and D_min_ are the maximum and minimum diameters respectively. The corrosion defect is also positioned symmetrically about YZ plane. The corroded pipeline’s diameter D is set to be 50.8mm. The pipeline’s full length 2L is set to be 4D. Mesh independency check has been passed by the grids in this paper: the number of elements in the axial direction is twelve both in corroded and intact regions and one element per 10° in the circumferential direction of the intact region with one element per 4° in the circumferential direction of corrosion region. Four elements are used in the radial direction of intact region. One element is used if d/t ≥ 0.7, while three elements are used if d/t ≤ 0.3, otherwise two elements are used in the radial direction of corroded region.

### 2.2 Boundary and load conditions

Due to symmetry, only a quarter of pipeline is modeled. The nodes at X = 0 and Z = 0 are set to be symmetric about YZ and XY plane respectively. The pipe end at Z = L is allowed to expand freely in the Z direction. Generally speaking, in ABAQUS there are two methods that could be used to calculate the collapse pressure: the arc-length method and volume controlled method. To use the latter method, an enclosed cavity should be created and the cavity will be filled with hydrostatic fluid elements F3D4. The pressure becomes an additional unknown parameter and the pressure loading procedure becomes volume controlled loading procedure. These two methods are essentially the same and will predict similar result [23]. In this paper, arc-length method is adopted. Tension load is applied at the end of pipeline and external pressure is applied on the external surface. Tension is loaded first and fixed at a specified value, and then the external pressure is applied until collapse. This loading sequence is represented by T → P.

### 2.3 Material properties

The material in this section is chosen to be stainless-steel 304. Ramberg-Osgood fit is chosen to represent the stress-strain relationship of stainless-steel 304, and its expression is given as:

$$\varepsilon =\frac{\sigma }{E}\left[1+\frac{3}{7}{\left(\frac{\sigma }{\sigma_{y}}\right)}^{n-1}\right]$$ (2)

In order for verification, the three parameters needed for the Ramberg-Osgood expression are kept the same with [22]: the Young’s modulus E = 191GPa, the effective yield stress σ_y_ = 212MPa, and the strain hardening exponent n = 9.

### 2.4 Verification of the FE model and mesh convergence check

**Fig 2:**
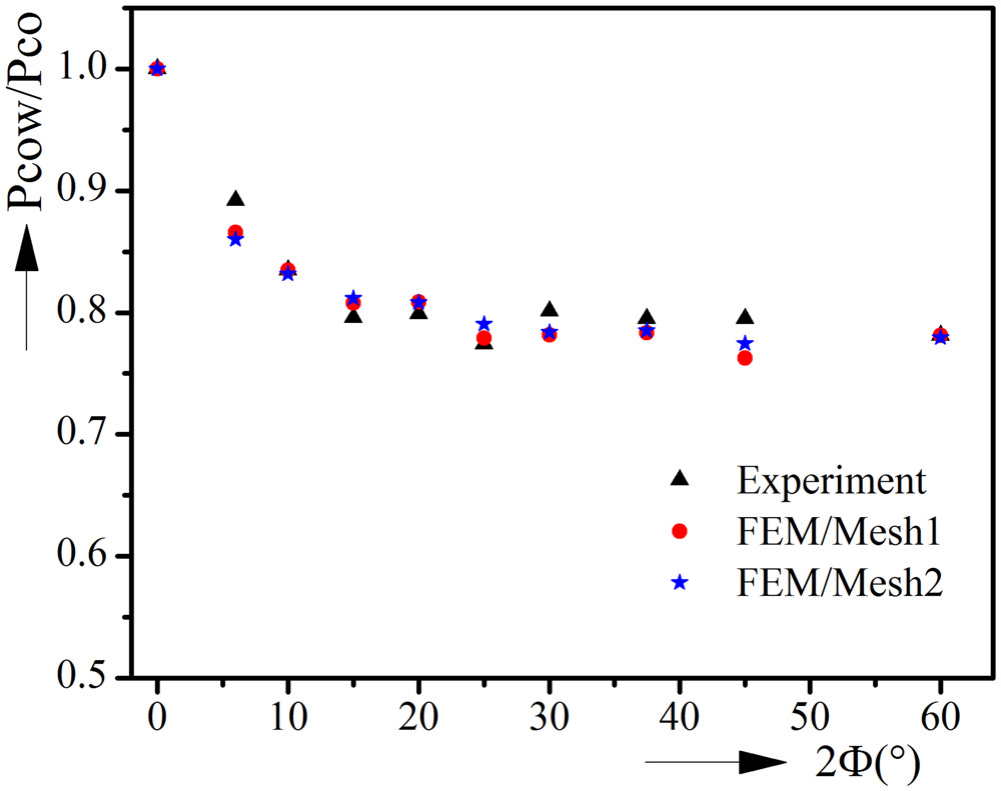
Comparison of collapse pressure between finite element analyses and experimental results.

The results of finite element method analysis are compared with existing experimental results [22]. In the experiments, internal corrosion defect was produced through wire electric discharge machine with a precision of 0.0254mm. The ovality orientation is symmetric about the symmetric plane of corrosion defect. A solid rod was placed through the pipeline, with each end connected with an end cap. Spaces were left between pipeline and end caps so that the axial loads caused by hydrostatic pressure would act on the rod, not the pipeline, and the pipeline could expand freely in the axial direction. The spaces were sealed using thin rubber tube with very thin Al foil layer under it. The influence of the thin Al foil layer on the collapse pressure was quite small and could be neglected after normalizing by collapse pressure of intact pipeline. The test specimens were placed in a pressure vessel with a pressure capacity of 69MPa. Then the vessel was filled with water and pressured under volume-controlled manner until the pipeline collapses. The geometric parameters of axially uniform corroded pipelines used in experiments are listed in Table 1. Fig 2 illustrates the experimental collapse pressure and the collapse pressure calculated by finite element method. The collapse pressure of corroded pipeline is normalized by the collapse pressure of intact pipeline. Pco is the collapse pressure of intact pipeline under pure external pressure, and Pcow is the collapse pressure of corroded pipeline.

**Table 1:** Geometric parameters of uniform corroded pipelines.

Model no.	D/t	Δ_0_(%)	d/t	2Φ (°)
1	20.99	0.19	0	0
2	21.01	0.17	0.256	6
3	21.01	0.29	0.259	10
4	21.10	0.22	0.262	15
5	20.96	0.36	0.257	20
6	21.10	0.25	0.265	25
7	21.11	0.21	0.263	30
8	21.10	0.20	0.255	37.5
9	21.05	0.13	0.259	45
10	20.97	0.33	0.265	60

The no.1 model is intact pipeline without corrosion; D/t is around the value of 21; Δ_0_(%) is ranging from 0.13 to 0.36; d/t is around the value of 0.26 (except for the intact pipeline model).

Mesh convergence check is carefully performed, see Fig 2. “Mesh1” represents the default mesh distribution explained in Section 2.1, while “Mesh2” represents a finer mesh distribution. The finer mesh adopts twice the element numbers used in default mesh distribution, i.e., 24 elements are used in the axial direction and one element per 5° and 2° in the circumferential direction of the intact region and corrosion region, respectively. It could be observed from Fig 2 that the differences of collapse pressure between the two mesh distributions are very small, which proves the validity of the default mesh distribution.

The comparison shows that the finite element model could predict the collapse pressure accurately. It could be seen from Fig 2 that the collapse pressure drops about 20% when corrosion angle increases from 0° to 15°. However, further increase of the corrosion angle from 15° to 60° has little effect on collapse pressure. The minor fluctuation of collapse pressure is caused by the fluctuation of geometry size in experiment [22].

### 2.5 Effect of tension on the collapse pressure

**Fig 3:**
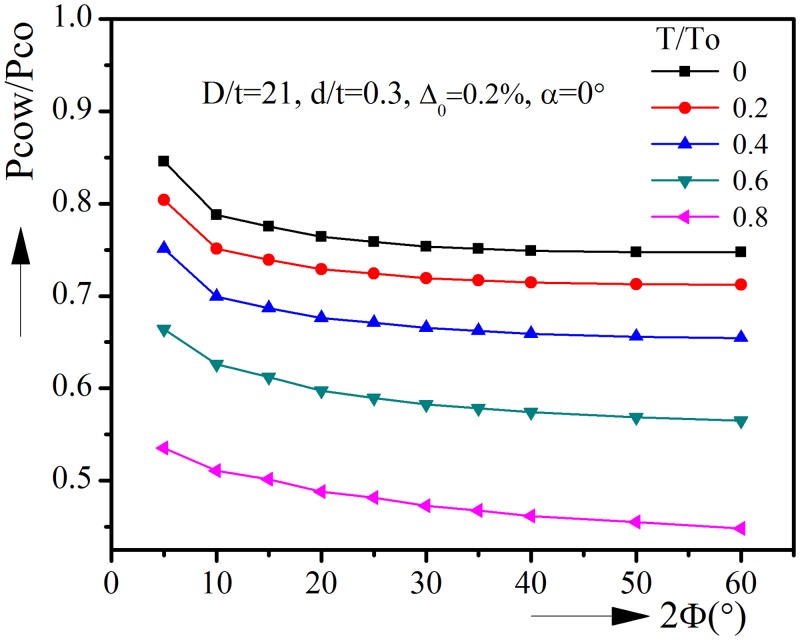
Collapse pressure vs. corrosion angle with various tensions.

In order to investigate the effect of tension on collapse pressure, a number of analyses are conducted with D/t = 21, d/t = 0.3 and Δ_0_ = 0.2%. Fig 3 shows that tension will affect collapse pressure significantly. The tension T is normalized by T_o_ = σ_o_A_o_ (σ_o_ is the yield stress, A_o_ is the cross section area of the corroded pipeline). It could be seen from Fig 3 that tension will generally reduce the collapse pressure for various corrosion angles. Take 2Φ = 20° for example, Pcow/Pco is 0.7642 when T/To = zero, but it reduces to 0.6766 when T/To = 0.4, and further reduce to 0.4882 when T/To = 0.8. Thus tension will reduce the collapse pressure significantly and should be taken into consideration when it exists.

**Fig 4:**
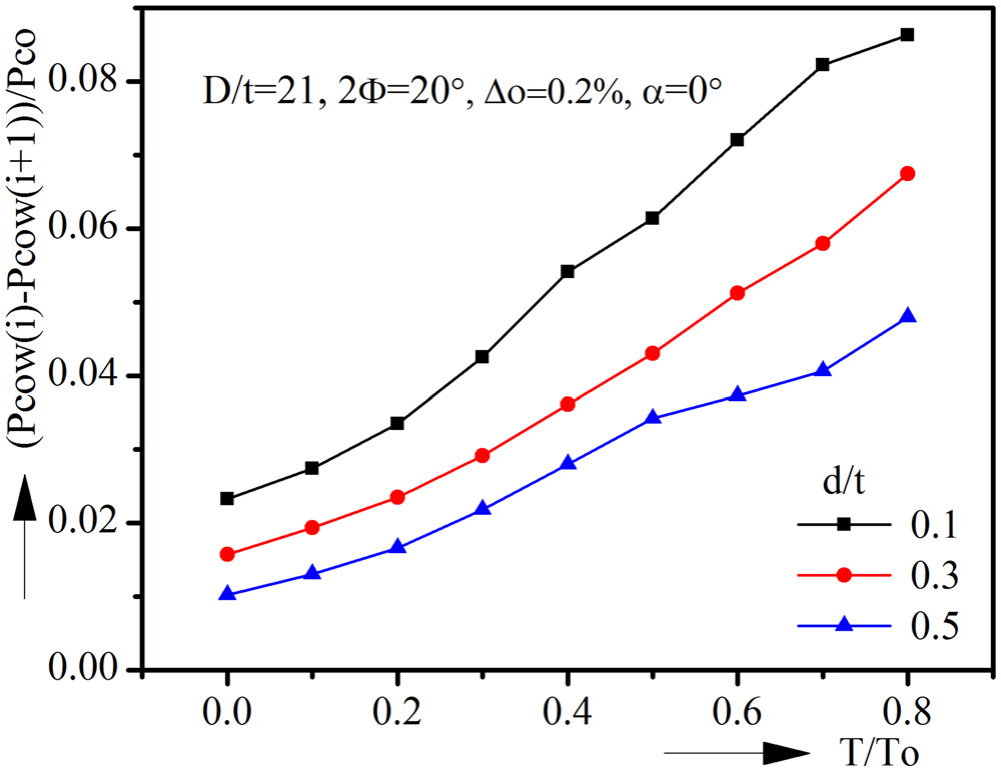
Collapse pressure decrement vs. tension with various corrosion depths.

Another phenomenon could be found is the decrement of collapse pressure tends to increase with the increase of tension. This could be seen more clearly by introducing the normalized collapse pressure decrement (Pcow(i)-Pcow(i+1))/Pco, where Pcow(i) is the collapse pressure with tension T/To(i) and Pcow(i+1) is the collapse pressure with tension T/To(i+1). Fig 4 shows the decrement pressure as the function of tension. As can be seen in the figure, when d/t = 0.1, the decrement of collapse pressure increases from 0.0232 to 0.0863 with tension increasing from 0 to 0.9. Notice that the corrosion defect with d/t = 0.1 is relatively shallow and when the corrosion defect becomes deeper, the decrement keeps increasing but being smaller than that of shallow ones.

### 3. Parametric Studies

It has been shown in Section 2 that the finite element results are in good agreement with experimental ones. From the above analyses it could be seen that tension is an important factor affecting the collapse pressure. Besides, several geometric parameters could affect the collapse behavior too, such as diameter-to-thickness ratio, initial ovality, corrosion angle, corrosion depth and ovality orientation. An extensive parametric study is conducted by using Python programming language to take a further look at how these parameters are affecting the collapse behavior under combined external pressure and tension. Since there are lots of models to be analyzed, to manually terminate every analysis when collapse pressure is reached would be very inconvenient. Thus a FORTRAN subroutine is written to automatically stop each analysis and record the result when collapse pressure is reached.

The pipeline material is now chosen to be API 5L Grade X52 [24], with Young’s modulus E = 207GPa and yield stress σ’_o_ = 359MPa at a strain of 0.5%. The stress at a strain of 0.2% is σ_o_ = 345MPa. Again, the material property is represented by Ramberg-Osgood fit and the hardening parameter n is set to be 13.6 since the influence of it on collapse pressure is relatively small for high values of n [3, 7]. 2C = 2Φ/360° is used to represent the corrosion angle. In this section, asymmetric collapse modes caused by ovality orientation are also investigated. The angle between the major axis of initial ovality imperfection and the symmetric plane of corrosion defect is α, ranging from 0° to 90°. The dimensionless form A = α/90° is used in the following. For pipeline with A = 0 and 1, the symmetric model used in the previous section is adopted, while for pipeline with 0 < A < 1, an asymmetric model will be used. The geometric parameters are listed in Table 2.

**Table 2:** Geometric parameters of axially uniform corroded pipelines.

Parameter	Value							
D(mm)	50.8							
D/t	10	15	20	25	30	35	40	50
d/t	0.1	0.2	0.3	0.4	0.5	0.6	0.7	0.8
Δ_0_(%)	0.2	0.3	0.4	0.5	0.6	0.7	0.8	0.9
	1.0	1.1	1.2					
2C	0.0222	0.0278	0.0333	0.0444	0.0555	0.0694	0.0833	0.0972
	0.1111	0.125	0.1389					
A	0	0.167	0.333	0.5	0.667	0.833	1	

### 3.1 Diameter-to-thickness ratio D/t

**Fig 5:**
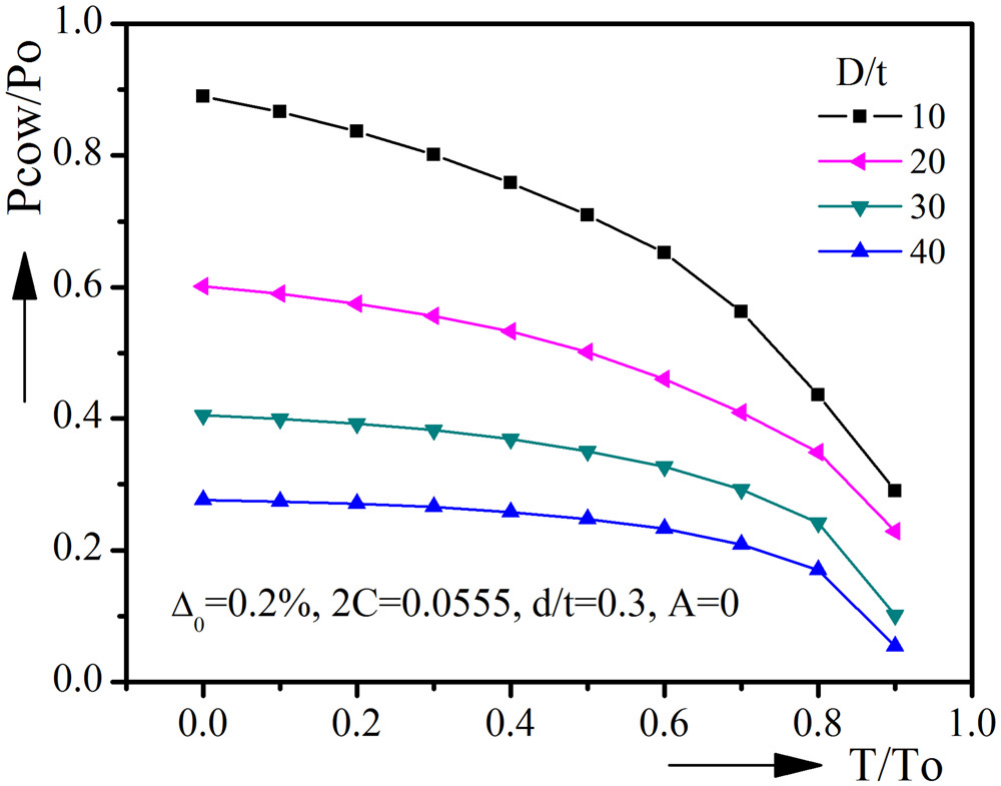
Effect of diameter-to-thickness ratio on collapse pressure-tension interaction curves.

Fig 5 shows the collapse pressure-tension interaction curves under various diameter-to-thickness ratios. Po is the yield pressure without tension, defined as Po = 2σ_o_t/D. The initial ovality, corrosion angle, corrosion depth and ovality orientation are kept constant in these analyses. In general the collapse pressure drops with the increase of D/t, but the decrement becomes smaller when D/t becomes larger. Take T/To = 0.5 for example, when D/t increases from 10 to 20, the decrement is 0.2077, while when D/t increases from 30 to 40, the decrement is 0.1031. In other words, relatively thin pipelines are less sensitive to the change of D/t. It’s worth pointing out that the collapse pressure of thin pipeline is much smaller than that of thick pipeline and couldn’t meet the requirement of deep water engineering. So the diameter-to-thickness ratio with D/t > 40 will not be included in Section 3.6’s empirical equation. As could be seen in Fig 5, tension will reduce the collapse pressure and the decrement increases with the increase of tension. Thus the curves are nonlinear and collapse pressure drops more quickly when tension increases, especially for very thick pipelines.

### 3.2 Initial ovality Δ_0_

**Fig 6:**
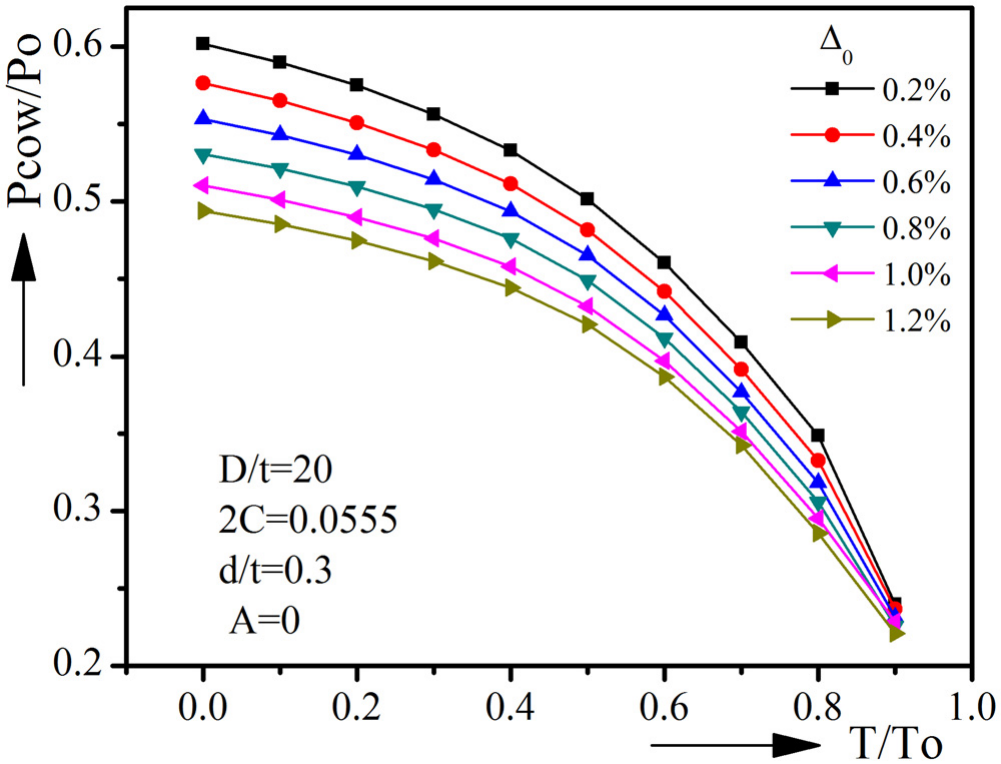
Effect of initial ovality on collapse pressure-tension interaction curves.

Initial ovality is an important factor which could detrimentally influence pipeline’s resistance to collapse. This influence is presented in Fig 6. All the cases herein are assumed to have the same diameter-to-thickness ratio, corrosion angle, corrosion depth and ovality orientation. When T/To = 0, the normalized collapse pressure drops from 0.6018 to 0.4939 with initial ovality increasing from 0.2% to 1.2%. The reduction becomes smaller when tension increases.

### 3.3 Corrosion angle Φ

**Fig 7:**
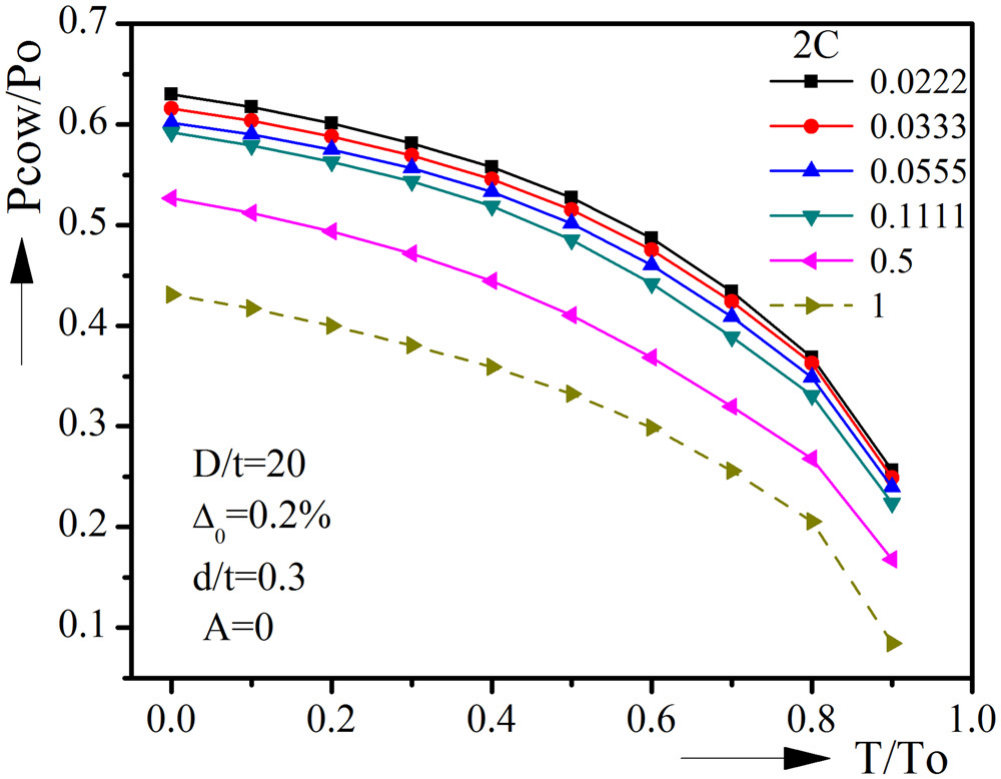
Effect of corrosion angle on collapse pressure-tension interaction curves.

The effect of corrosion angle on collapse behavior under combined external pressure and tension is presented in Fig 7. The diameter-to-thickness ratio, initial ovality, corrosion depth and ovality orientation are kept constant in these analyses. The collapse pressure with corrosion angle of 0.5(180°) and 1(360°) are also included for comparison. The dashed line is the lower bound of the collapse pressure-tension interaction curves with various corrosion angles. It is seen that the increase of corrosion angle will reduce the collapse pressure. When T/To = 0.5, the collapse pressure reduces from 0.5271 to 0.4104 when corrosion angle increases from 0.0222 to 0.5 and it will further reduce if the corrosion angle further increases.

### 3.4 Corrosion depth d/t

**Fig 8:**
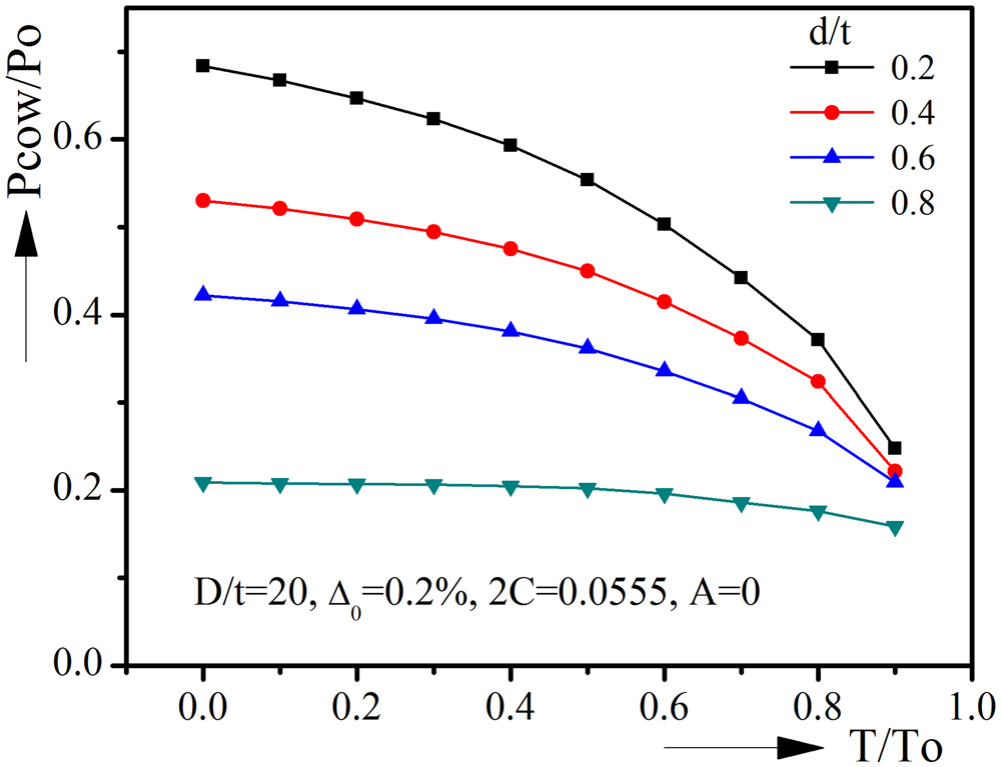
Effect of corrosion depth on collapse pressure-tension interaction curves.

Fig 8 shows the collapse pressure-tension interaction curves for various corrosion depths. The diameter-to-thickness ratio, initial ovality, corrosion angle and ovality orientation are kept constant in these analyses. The collapse pressure drops significantly with the increase of corrosion depth. When there is no tension, Pcow/Po is 0.6833 with d/t = 0.2, and it drops to 0.2089 with d/t = 0.8. For all range of d/t, the collapse pressure decreases with the increase of tension, however, the decrement becomes relatively small when d/t reaches 0.8. When d/t = 0.8, the decrement of Pcow/Po from T/To = 0 to T/To = 0.9 is 0.0503, while the corresponding decrement is 0.436 when d/t = 0.2. It is worth pointing out that deep corrosion will reduce the pipeline’s resistance to collapse severely and should be prevented. The corrosion depth d/t > 0.6 will not be taken into account in the empirical equation.

### 3.5 Ovality orientation α

The previous parametric studies are based on symmetric models, with the corrosion defect be symmetric about the major axis of ovality imperfection. However, the corrosion defect could occur at arbitrary position during operation and the angle A(α) would vary from 0(0°) to 1(90°). Thus asymmetric collapse modes caused by asymmetric imperfections should also be analyzed.

**Fig 9:**
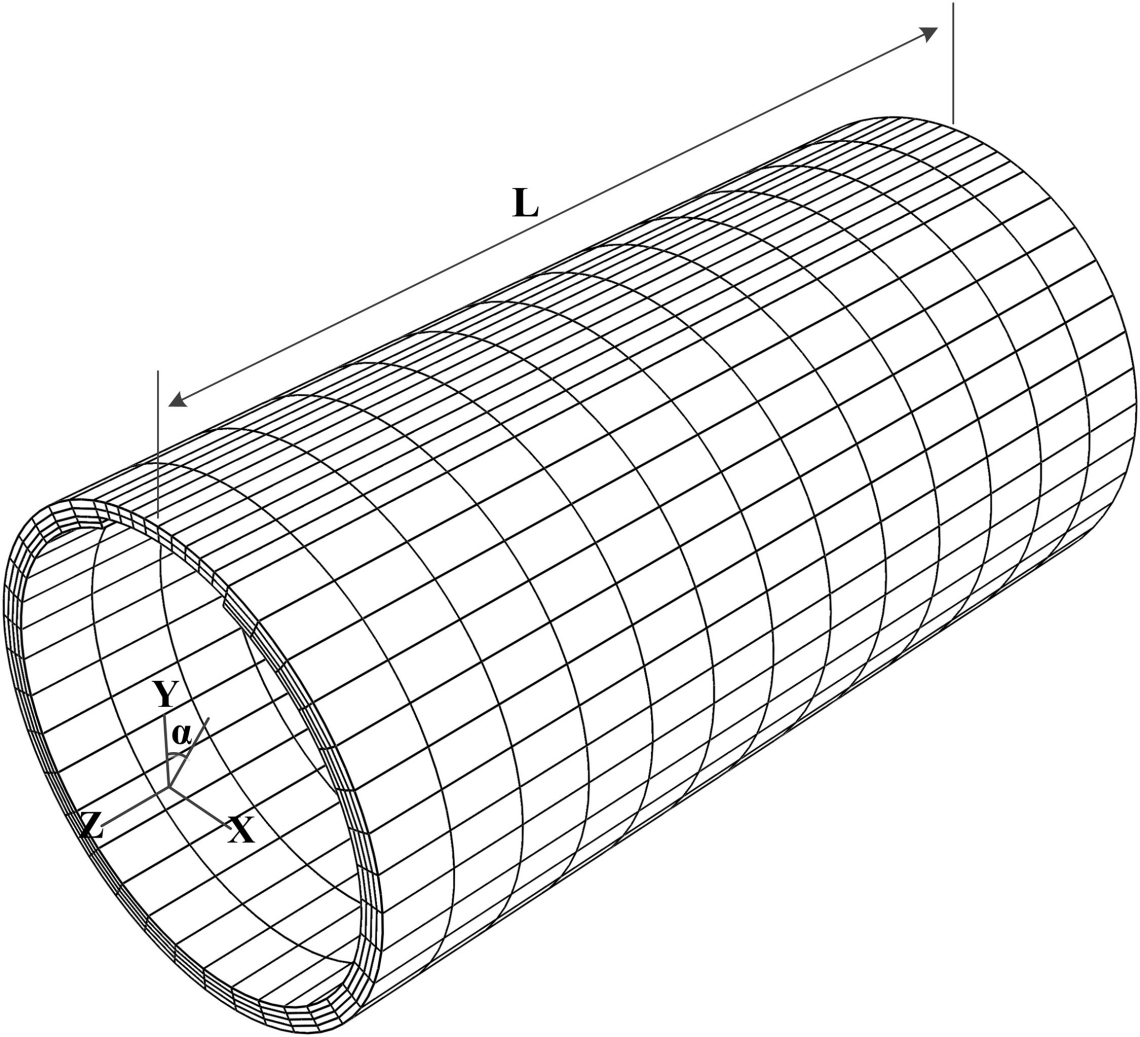
Finite element model of pipelines with asymmetric initial ovality imperfection.

Because of asymmetry, one half of the corroded pipeline is modeled, see Fig 9. The nodes at Z = 0 are set to be symmetric about XY plane. Instead of rigid constraints, which might cause stress concentration in the asymmetric collapse mode, spring constraints are used to prevent rigid body movement. The mesh distribution and the load conditions are similar to the symmetric model.

**Fig 10:**
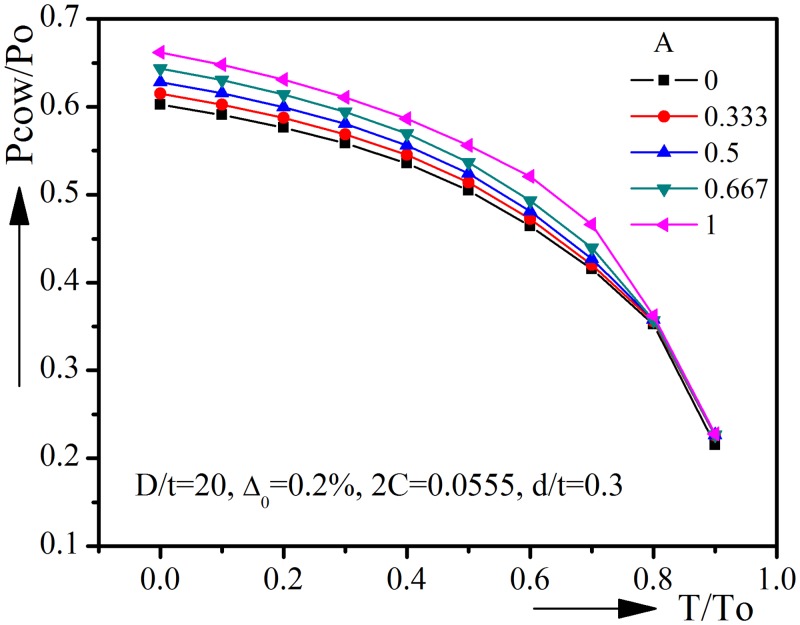
Effect of ovality orientation on collapse pressure-tension interaction curves.

The effect of ovality orientation on collapse pressure is shown in Fig 10. It could be seen that ovality orientation has certain effect on the collapse pressure, especially when T/To is less than 0.8. For each value of T/To, the collapse pressure of A = 0 is the lowest one among others. This is the position where the major axis of ovality imperfection coincides with the symmetric plane of corrosion defect thus the influence on collapse behavior of each is additive. With the increase of A, the additive effect is reduced and the collapse pressure increases. For T/To = 0, the collapse pressure is 0.6024 when A = 0, and it increases to 0.6619 when A = 1, the difference of collapse pressure between A = 0 and A = 1 is 0.06. However, the difference becomes much smaller when T/To > 0.7. For T/To = 0.8, the collapse pressure of A = 0 and A = 1 are 0.3623 and 0.3521 respectively, with a difference of 0.01.

### 3.6. Empirical equation

The results of parametric studies are now used to develop an empirical equation of the interaction of external pressure and tension on collapse of axially uniform corroded pipeline.

First, considering the collapse of intact pipeline under pure external pressure, this equation can be written in the following form:

Pco/Po=f(D/t,Δo) (3)

Then, considering the collapse of corroded pipeline under pure external pressure, a modified equation is given by including the corrosion defect parameters:

Pcow/Po=f(D/t,Δo)⋅[1−g(D/t,Δo,2C,d/t,A)] (4)

Finally, by taking account of the detrimental effect of tension, the final form of empirical equation is given as:

Pcow/Po=f(D/t,Δo)⋅[1−g(D/t,Δo,2C,d/t,A)]⋅h(D/t,Δo,2C,d/t,A,T/To) (5)

After several attempts, each part of Eq (5) could be represented as follows:

$$f(D/t,\Delta o)=3.783\text{-}1.615{\left(D/t\right)}^{0.19}-0.26{\left(\Delta o\right)}^{0.421},$$

$$g(D/t,\Delta o,2C,d/t,A)=\frac{{\left(D/t\right)}^{0.443}{\left(\Delta o\right)}^{\text{-0.275}}{\left(2C\right)}^{0.356}{\left(d/t\right)}^{1.744}[1.457-0.771{\left(A\right)}^{2.11}]}{0.544+1.524{\left(D/t\right)}^{0.458}{\left(\Delta o\right)}^{\text{-0.282}}{\left(2C\right)}^{0.374}{\left(d/t\right)}^{0.968}},$$

h(D/t,Δo,2C,d/t,TTo,A)=(0.337−1.154(2C)0.036+1.191(d/t)0.0834.092−0.003(D/t)1.932(Δo)0.371(A)0.001)^[0.088(TTo)+0.458(TTo)3.618].

**Fig 11:**
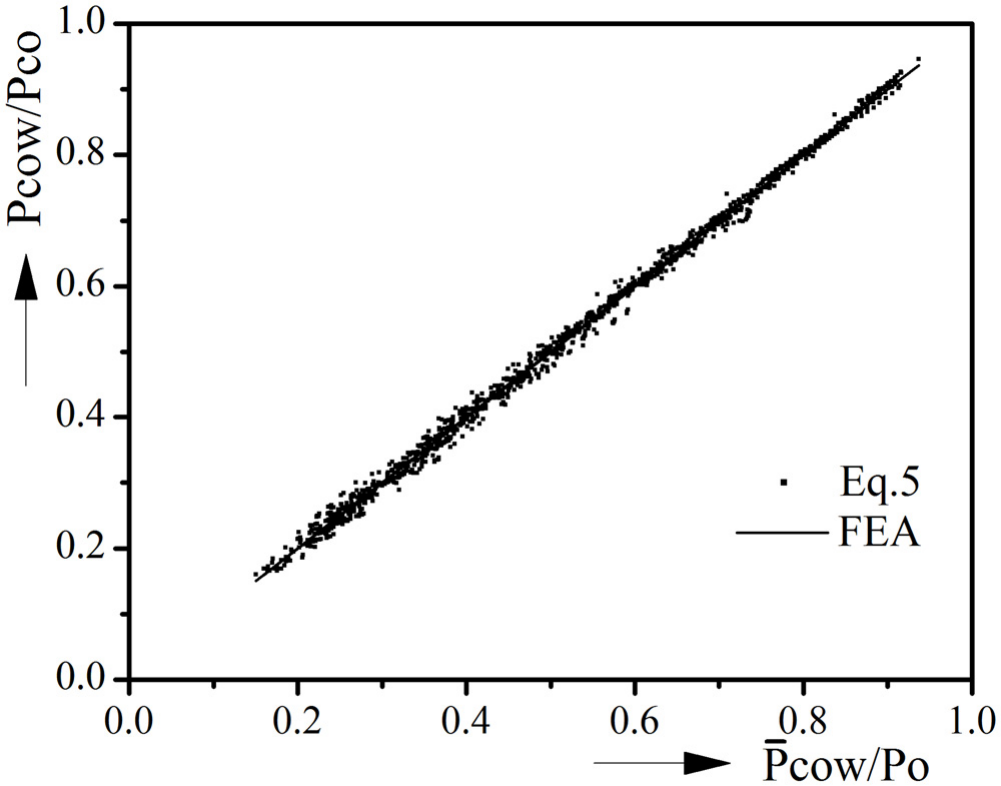
Comparison between collapse pressures from Eq 5 (Pcow) and numerical results (P̅cow).

Fig 11 shows the comparison between collapse pressures from Eq 5 and the finite element analyses. It could be seen that the collapse pressures predicted from the equation are in good agreement with numerical ones. It is worth noting again that pipelines with D/t > 40 or d/t > 0.6 have relatively small collapse resistance, thus they are not suitable for deep sea exploration and will not be used to generate the empirical equation. The empirical equation would be expected to work within the following parameters range: 10 ≤ D/t ≤ 40, 0.2 ≤ Δ_0_ (%) ≤ 1.2, 0.0222 ≤ 2C ≤ 0.1389, 0.1 ≤ d/t ≤ 0.6, 0 ≤ A ≤ 1. Besides, this empirical equation is only suitable for materials with similar stress-strain relationship as described before.

## 4. Additional Parametric Studies

### 4.1 Loading path

The above sections are analyzing under the T → P loading. However, during installation and operation, offshore pipelines may undergo different loading paths, such as the P → T loading. Thus it is worth analyzing the influence of different loading paths on collapse behavior. We consider pipelines with D/t of 20 and 30 while keeping the other geometric parameters fixed. Without loss of generality, the symmetric model of A = 0 is used. In addition, the material properties of all pipelines are kept the same as before.

**Fig 12:**
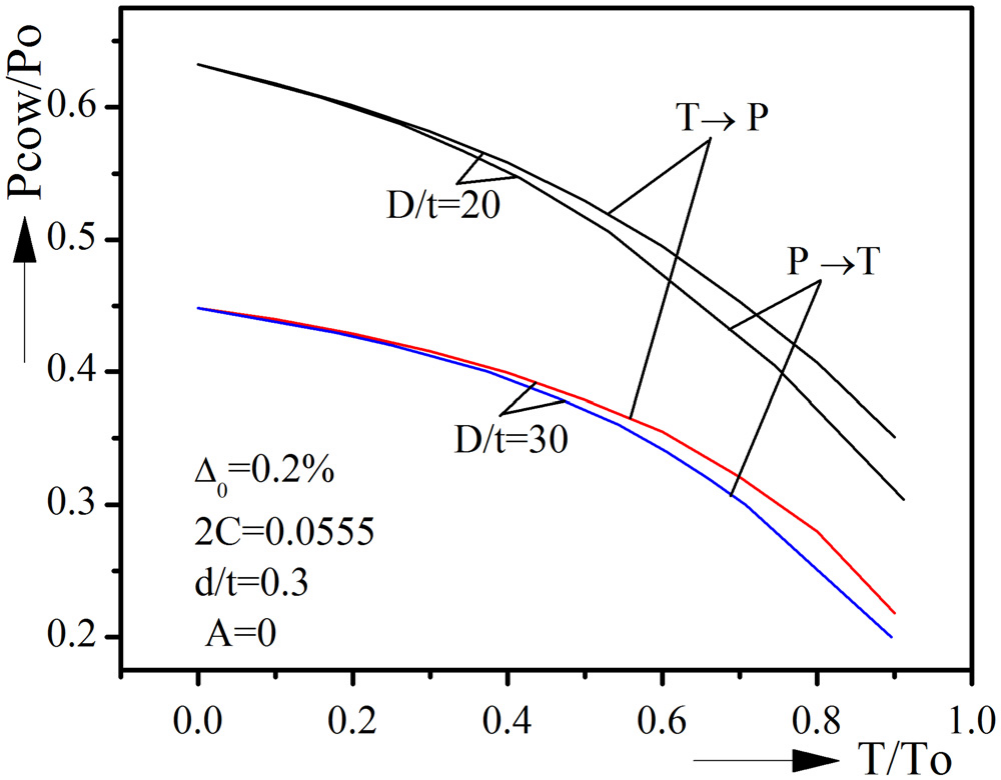
Influence of loading path on collapse behavior for D/t = 20 and 30.

Under the P → T loading, pipeline is first loaded under pure external pressure and fixed at a given value, then tension is applied until collapse happens. The interaction curves for D/t = 20 and 30 under P → T loading are shown in Fig 12, also included in the figure are the corresponding curves for T → P loading path.

It could be seen that loading path will affect the collapse behavior. Pipelines under P → T loading have smaller collapse pressures in general. Nevertheless, the difference between two loading paths remains very small and could be ignored if T/To is less than 0.5. When tension is larger, the difference becomes larger and the influence of loading path should not be ignored.

### 4.2 Initial imperfection length

Although the influence of initial imperfection with the form of ovality has been discussed, all those analyses are based on the assumption that ovality is axially uniform. Actually the initial imperfection could have varied length. This part aims at researching into the problem whether the length of initial imperfection will affect the collapse behavior under combined external pressure and tension.

**Fig 13:**
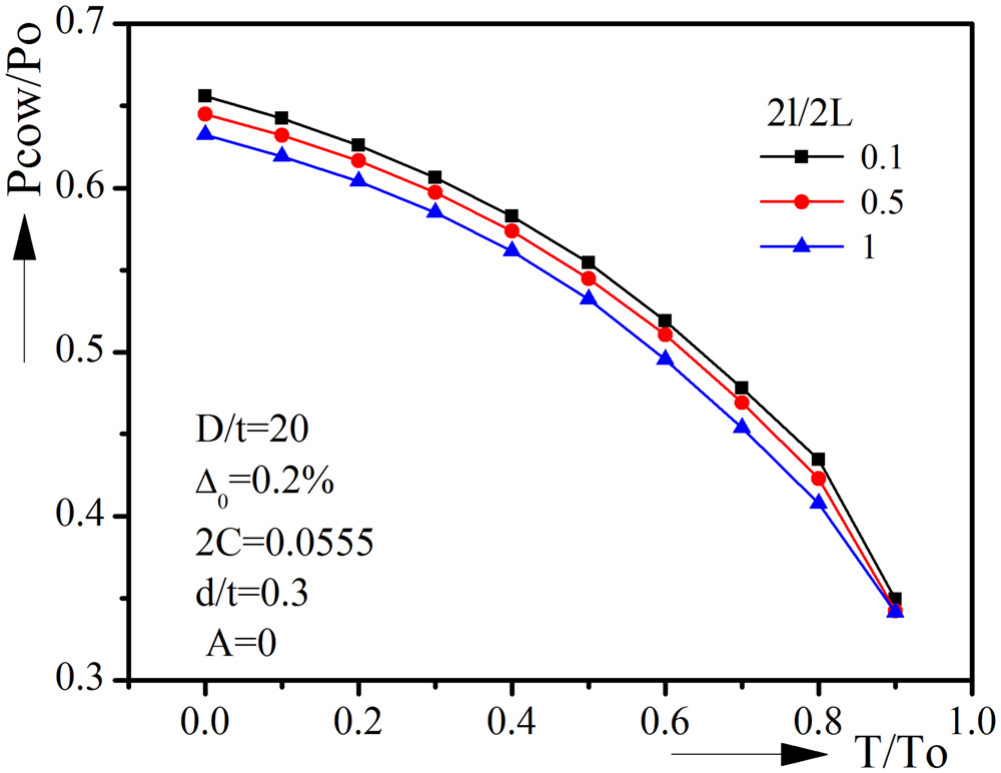
Influence of initial imperfection length on collapse behavior under combined external pressure and tension.

The D/t, Δ_0_, 2C, d/t and A are kept constant in the following analyses. The initial imperfection is defined in the following form:

$$w=\{\begin{array}{c} \Delta oR\text{cos}2\theta \;for\;0\leq z\leq l\\ \Delta oR\frac{l+\alpha -z}{\alpha }\text{cos}2\theta \;for\;l\leq z\leq l+\alpha \\ 0\;for\;l+\alpha \leq z\leq L \end{array}$$ (6)

where 2*l* is the initial imperfection length, α is the length of the transition region between perfect and imperfect regions. Here α is set to be 0.2D. The results are shown in Fig 13.

It could be seen that the length of initial imperfection will affect the collapse pressure to some extent, but the influence is relatively small. For very high tension, say T/To = 0.9, the influence is neglectable. When we use the axially uniform ovality assumption in design, we will always be on the safe side.

### 4.3 Anisotropic yielding

Anisotropic yielding is often induced by pipeline manufacturing process. The effect of yield anisotropy should also be determined. In this work, Hill anisotropy yielding function [25] is used to model the yielding of pipelines. An appropriate form of the function could be written in terms of cylindrical coordinate as

$$\sigma_{oz}={\left[\sigma_{z}^{2}-\left[1+\frac{1}{S_{\theta }^{2}}-\frac{1}{S_{r}^{2}}\right]\sigma_{z}\sigma_{\theta }+\frac{1}{S_{\theta }^{2}}\sigma_{\theta }^{2}\right]}^{1/2}$$ (7)

where S_θ_ = σ_oθ_ / σ_oz,_ S_r_ = σ_or_ / σ_oz_, and σ_or_, σ_oθ_, σ_oz_ are yielding stresses in the radial, circumferential and axial directions. Since the work presented here is to have a preliminary look into the effect of anisotropic yielding on the collapse pressure under combined loads, it is convenient to assume S_θ_ = S_r_ = S. Actually this has been adopted by many researchers [5, 10, 23].

**Fig 14:**
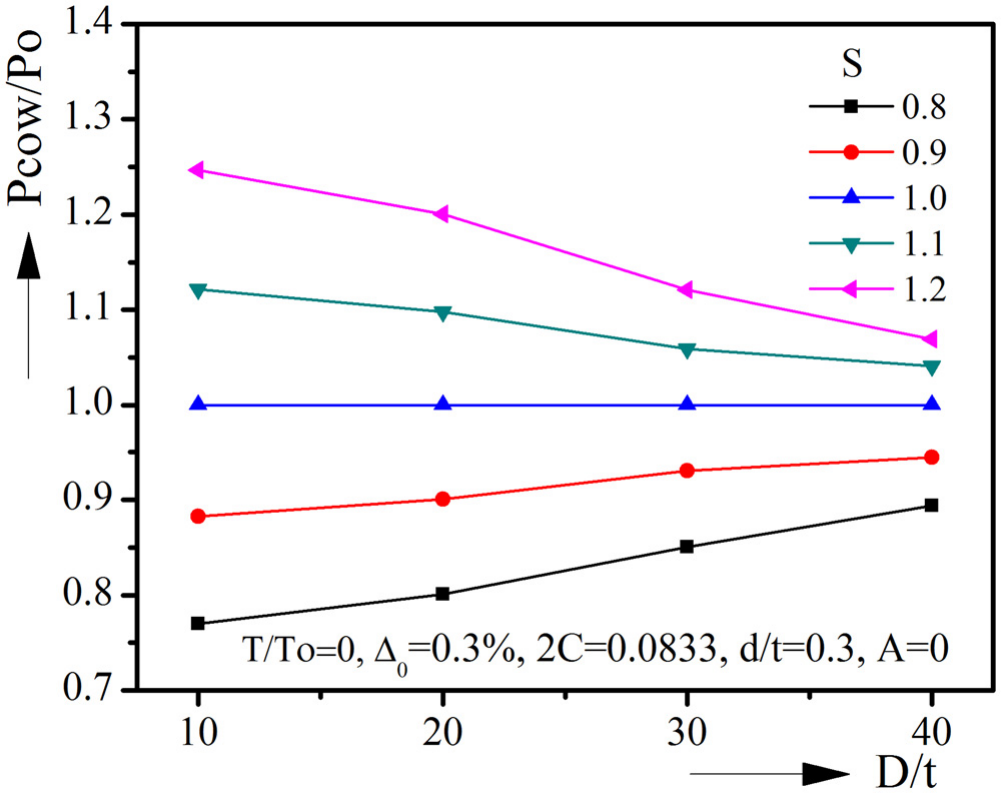
Effect of yield anisotropy on collapse pressure under combined loads.

The effect of anisotropy is shown in Fig 14. The collapse pressure is normalized by the P’cow, which is the collapse pressure of corroded pipeline when S = 1. S < 1 leads to a decrease of collapse pressure, and S>1 leads to an increase in collapse pressure. From Fig 14 we can see that the influence of anisotropy on the collapse pressure becomes smaller for high D/t pipelines, which tend to buckle in the elastic range.

### 4.4 Corrosion defect length

**Fig 15:**
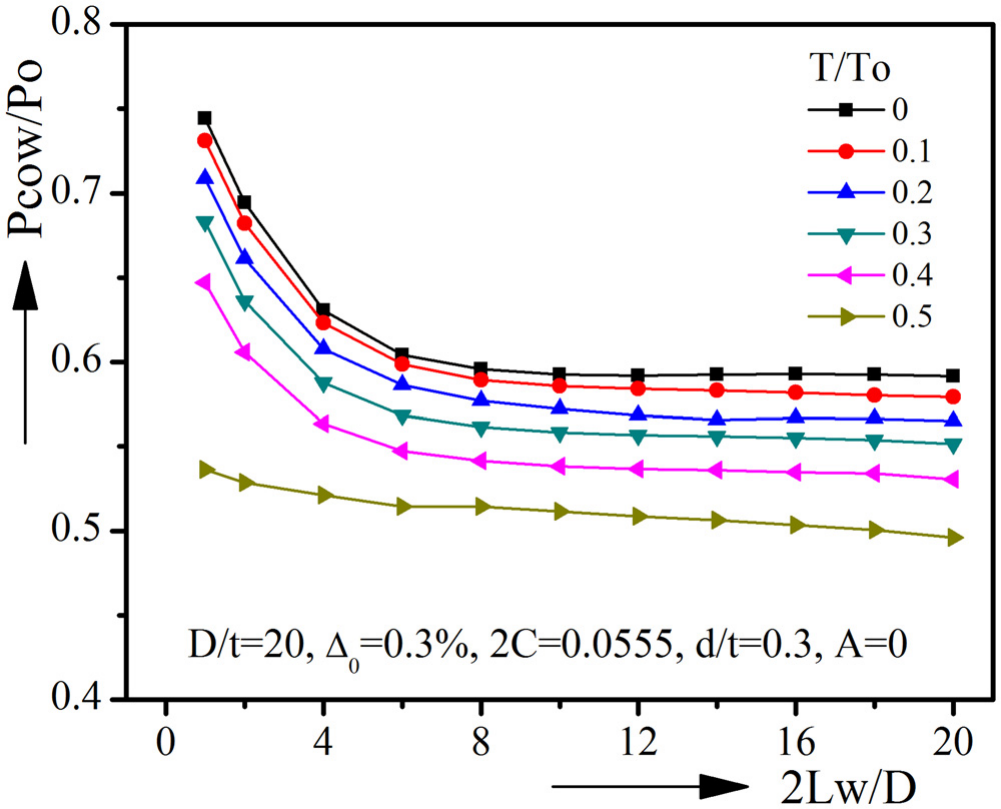
Effect of corrosion defect length on collapse pressure under varied tension (D/t = 20).

Long and short longitudinal corrosion defects could both occur in subsea pipelines. Since the above sections are all dealing with long longitudinal corrosion defects, it is also worth finding out how the length of corrosion defect will affect the collapse pressure under combined loads. For this purpose, a second pipeline model with corrosion defect running through part of the length is created. The pipe length 2L is set to be 20D, the corrosion defect length 2Lw is varied from 1D to 20D. The material and boundary condition are similar to those used in Section 3. Corroded pipelines with different geometric parameters are analyzed. Fig 15 plots the normalized collapse pressure against corrosion defect length under different tension for pipelines with D/t = 20, Δ_0_ = 0.3%, 2C = 0.0555(20°), d/t = 0.3, A = 0(0°). Take T/To = 0 for example, with the increase of defect length, the collapse pressure drops quickly at the first stage. After 2Lw/D reaches 8, a further increase of defect length has almost no effect on the collapse pressure and the collapse pressure finally reaches to the value of 2Lw = 20D. Note that 2Lw = 20D is the case of axially uniform corroded pipeline. Similar trends could be found for pipelines under larger T/To.

**Fig 16:**
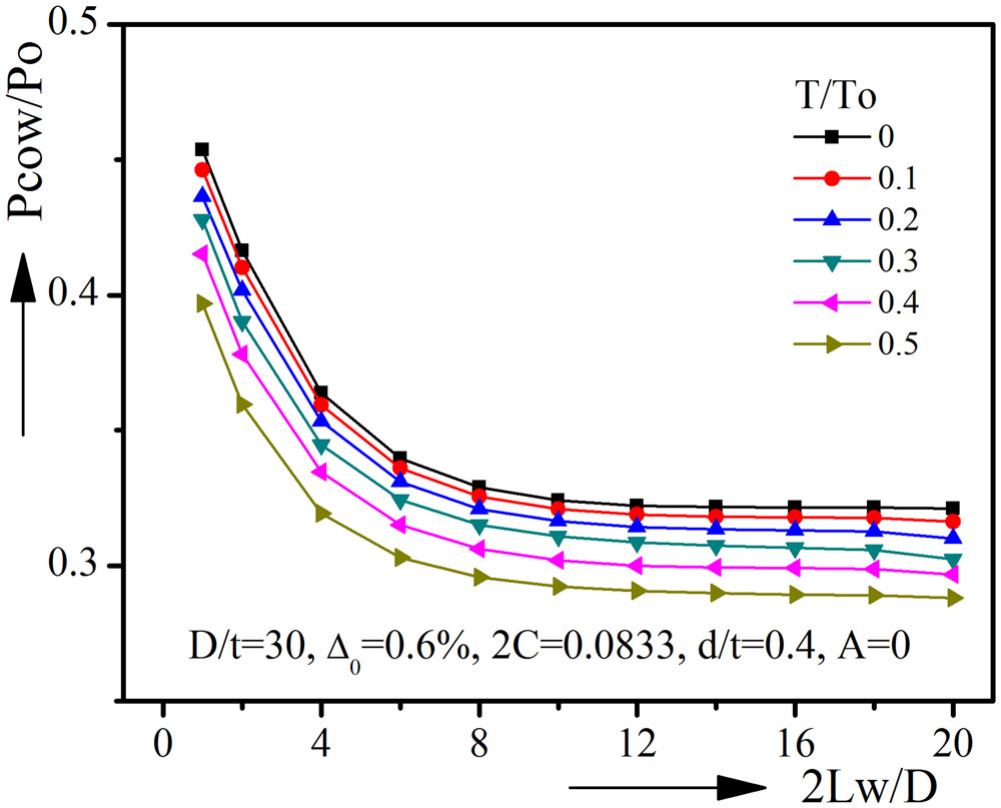
Effect of corrosion defect length on collapse pressure under varied tension (D/t = 30).

The results for pipelines with D/t = 30, Δ_0_ = 0.6%, 2C = 0.0833(30°), d/t = 0.4, A = 0(0°) are shown in Fig 16. Once again, the similar trends found in Fig 15 could be seen in Fig 16. When corrosion defect length 2Lw/D is larger than 10D, the collapse pressure will no longer be affected by the increase of defect length. Corroded pipelines of other geometric parameters are analyzed, and all show the similar trends. It could be concluded that pipelines with short longitudinal corrosion defects should be modeled as partial corroded pipelines, while pipelines with longer corrosion defect, such as 2Lw ≥ 10D, could be modeled as axial uniform corroded pipelines.

## 5. Conclusions

The problem of collapse of axially uniform corroded pipeline under combined external pressure and tension has been studied. Finite element analysis is used in this paper and its validity is first verified with existing experimental results. Extensive parametric studies are conducted in order to investigate the influence of geometric parameters on collapse behavior. The conclusions are listed as follows:

1. Tension reduces the collapse pressure for all parameters range. The decrement of collapse pressure increases with the increase of tension.
2. Diameter-to-thickness ratio, initial ovality, corrosion angle, corrosion depth and ovality orientation could significantly influence the collapse behavior under combined loads. Pipelines with large D/t or deep corrosion defect should be avoided in deep sea pipeline engineering since they have relatively small collapse resistance.
3. The empirical equation for predicting the collapse pressure under tension, Eq 5, is obtained based on the results of parametric studies. The predicted collapse pressures are in good agreement with numerical ones. The equation could be used to predict collapse pressure for a wide parameter range of practical pipelines.
4. Anisotropic yielding could have positive or negative effect on the collapse pressure under combined loads, depending on the anisotropy variable S. Pipelines with relatively short corrosion defect should be modeled with actual defect length, while for those with long corrosion defect it is conveniently to modeled as axially uniform corroded pipelines and the collapse pressure could be conveniently estimated using Eq 5. The influence of loading path on the collapse pressure increases with the increase of tension. Initial imperfection length has relatively smaller influence on collapse behavior under combined loads.
